# A computational approach to optimising laccase-mediated polyethylene oxidation through carbohydrate-binding module fusion

**DOI:** 10.1186/s12896-023-00787-5

**Published:** 2023-07-06

**Authors:** Michael Gollan, Gary Black, Jose Munoz-Munoz

**Affiliations:** Department of Applied Sciences, Northumbria University, Newcastle Upon Tyne NE1 8ST, Tyne and Wear, England, United Kingdom

**Keywords:** Polyethylene, Oxidation, Bioremediation, Optimisation, Directed evolution, Protein engineering, Laccase

## Abstract

Plastic pollution is a major global concern to the health and wellbeing of all terrestrial and marine life. However, no sustainable method for waste management is currently viable. This study addresses the optimisation of microbial enzymatic polyethylene oxidation through rational engineering of laccases with carbohydrate-binding module (CBM) domains. An explorative bioinformatic approach was taken for high-throughput screening of candidate laccases and CBM domains, representing an exemplar workflow for future engineering research. Molecular docking simulated polyethylene binding whilst a deep-learning algorithm predicted catalytic activity. Protein properties were examined to interpret the mechanisms behind laccase-polyethylene binding. The incorporation of flexible GGGGS(x3) hinges were found to improve putative polyethylene binding of laccases. Whilst CBM1 family domains were predicted to bind polyethylene, they were suggested to detriment laccase-polyethylene associations. In contrast, CBM2 domains reported improved polyethylene binding and may thus optimise laccase oxidation. Interactions between CBM domains, linkers, and polyethylene hydrocarbons were heavily reliant on hydrophobicity. Preliminary polyethylene oxidation is considered a necessity for consequent microbial uptake and assimilation. However, slow oxidation and depolymerisation rates inhibit the large-scale industrial implementation of bioremediation within waste management systems. The optimised polyethylene oxidation of CBM2-engineered laccases represents a significant advancement towards a sustainable method of complete plastic breakdown. Results of this study offer a rapid, accessible workflow for further research into exoenzyme optimisation whilst elucidating mechanisms behind the laccase-polyethylene interaction.

**Supplementary Information:**

The online version contains supplementary material available at 10.1186/s12896-023-00787-5.

## Introduction

Global plastic dependence and concurrent production has been increasing since its commercialisation in the 1950’s. As of 2015, an estimated 8,300 million tonnes have been produced, with approximately 7% recycled and the remainder either incinerated (10%) or disposed of within landfills or the environment (60%) [1]. Rising production rates are necessary to satisfy demand, with a total of 12,000 million tonnes of plastic predicted to have been disposed of by 2050 [1]. Polyethylene (PE) is the most abundant plastic produced owing to its strength, flexibility, and longevity. It is also, therefore, the most common pollutant [1–4], followed by polypropylene and polystyrene [5, 6]. PE is either low-density (LDPE) or high-density (HDPE), with the former containing periodically branched methylene chains which reduces its compactness and thus density, strength, and recalcitrance to degradation. Plastic pollution is ubiquitous, even within remote regions relatively spared of direct anthropogenic influence, such as the French Pyrenees [7] and Mount Everest [8]. Indeed, through the natural breakdown of macroplastics (MaPs), secondary microplastics (5 mm-1 μm, MPs) and nanoplastics (< 1 μm, NPs) are generated, with the latter susceptible to relatively limitless atmospheric dispersal. Owing to their size, both MPs and NPs are thus susceptible to meteorological events, observing seasonal-dependent deposition and environmental flux [2, 9]. Whilst marine plastics receive greater attention due to ubiquity, biological detriment, and large-scale dispersal [10], terrestrial pollution is also highly prevalent [4, 11]. Increasing agricultural land-usage, coupled with concurrent plastic dependence, yields significant soil deposition; for example, the use of polyethylene-based mulch for soil aeration [12].

Through ingestion and entanglement [13, 14], polluted plastics severely impact animal abundance and diversity. This extends to humans, with MPs found within common consumables [5, 6, 13, 15, 16] and in the air [16], yielding an estimated 30,000 to 140,000 MPs consumed per individual per annum [16]. Whilst the physiological effects of plastic ingestion are poorly understood, research suggests the potential for dissemination from both digestive (ingested) [17] and respiratory (inhaled) tissues [18, 19] and consequent deposition within distal areas. Recent experiments indicate developmental [20, 21] and oncogenic/mutagenic [19, 20, 22] consequences of MP exposure, with potential neurological [15], endocrine [15, 19, 23], hepatic, renal [19], and immunological [15, 17, 18, 23] involvement.

As recognised within global sustainability targets [24], plastic waste management is thus of utmost importance for limiting environmental exposure. Our current methods for plastic removal are, however, inefficient. Complete breakdown through incineration generates significant carbon and toxic emissions, whilst recycling practices serve to only prolong inevitable disposal due to reduced strength and thus value of resulting secondary plastics [1]. Bioremediation of waste plastics is an attractive alternative. Microbial assimilation of plastic hydrocarbons favours complete degradation and bears biovalorisation potential through the production of valuable products [25]. Of the four stages of bioremediation: biodeterioration; biofragmentation; assimilation; and mineralisation, the initial two are pivotal for permitting microbial plastic uptake. Indeed, depolymerisation and oxidation of the hydrocarbon backbone are a necessity, with oligomers of ~ 10–55 carbons permissible for microbial uptake [13, 14]. These processes, however, are currently inefficient for large-scale implementation. The rate-limiting biodeterioration and biofragmentation processes suffer from the recalcitrance of PE surface hydrophobicity and the consequently slow enzymatic oxidation [12, 15]. Exoenzymes, secreted from plastic-adhered microorganisms, perform this initial biodeterioration [13, 16, 20], with lipases, peroxidases, and laccases observing increased expression and activity upon PE exposure [17–19, 26, 27]. Demonstrating higher enzymatic activity [22] and concurrent degradation rates [21], laccases are key mediators of PE biodeterioration. However, the enzymatic rate of depolymerazion is very slow, due to the stability of the C-C linkage present in PE. Laccases are multicopper oxidases (MCOs) involved in multiple enzymatic processes such as lignin depolymerization, sporulation, pigment production, and transforming phenols into oxidized quinones with the reduction of molecular oxygen [23]. However, the exact mechanism of laccase-mediated PE degradation is not fully understood.

Despite a necessity for improved oxidation and depolymerisation rates, exoenzyme engineering research is relatively sparse. Recently, Dai et al. [28] demonstrated the viability of this approach, with enhanced polyethylene terephthalate (PET) degradation achieved through fusing a carbohydrate-binding module (CBM) domain to PET hydrolase. CBMs are classified based on type and family. The former reflects substrate binding properties whilst the latter groups CBMs by sequence homology, often also reflecting substrate and binding similarities. Type A CBMs possess a characteristic planar binding site conformation, generally composed of three aromatic amino acid residues. This facilitates binding to similarly planar, crystalline, hydrophobic surfaces, such as cellulose. Types B and C CBMs juxtapose this type A conformation, binding glycans within tertiary folds and clefts forming more typical binding sites. A type A CBM family 1 (CBM1) domain was selected to enhance PET binding due to similar crystallinities observed between PET and cellulose, its native substrate. The planar hydrophobic surface common to type A CBMs is proposed to mediate this hydrophobic interaction, facilitating attachment to crystalline regions of target molecules. Indeed, both PET and cellulose binding has been reported by other type A CBMs [24], but not by type B or C [25].

As both PET and PE share similar semi-crystalline surfaces, we hypothesized that type A CBMs may facilitate laccase binding and thus oxidation of PE. This has not previously been explored. In this study, a bioinformatic approach was employed to predict the enzymatic properties of two type A CBM family (CBM1 and CBM2) laccase chimeras in the binding and catalysis of dodecane and eicosane, as representatives of short-chain PE hydrocarbons [21, 29]. Novel, putative, laccases were selected as candidates through genomic mining of known petrochemical-utilisers [28, 30–37] and those investigated within our research group: species belonging to the *Fusarium*, *Stenotrophomonas*, and *Salipaludibacillus* genera. Protein properties were examined to extrapolate the mechanism of action behind predicted enzyme kinetics, offering rationale for future directed evolution research.

## Results

### Selection of candidate laccases

An in-silico approach was undertaken to screen several laccase candidates. Supplementary data from Zampolli et al. [27] reported a significantly upregulated multicopper oxidase (GenBank Accession: AII08809.1) in *Rhodococcus opacus* cultivated on PE. This was used as a query for genome mining of candidate laccases, using Protein Basic Local Alignment Search Tool (BLASTp). Two putative MCOs were identified within *Stenotrophomonas acidaminiphila*, with relatively low identity scores (29.88%) but high coverage (84%). Predicted twin-arginine translocation (TAT) signal peptides supports their potential role as native exoenzymes to permit petrochemical utilisation by *S. acidaminiphila*. Similar to that performed by Santacruz-Juarez et al. [38], preliminary molecular docking between dodecane and MCOs was performed to investigate protein-ligand interactions. A laccase of *Trametes versicolor* was included as a positive control with experimentally confirmed PE binding [38]. The resulting change in Gibbs free energy (ΔG) reflected predicted PE binding affinity, with both putative *S. acidaminiphila* MCOs returning lower predicted binding energies than the positive control. One MCO, hereafter referred to as MCO1 (GenBank Accession: WP_182335395), returned a lower ΔG (-5.2) than both the positive control (-4.3) and the other MCO (MCO2, -4.6). MCO1 was thus selected as a suitable candidate for protein engineering. The genome of *F. venenatum* was also explored, encoding 13 putative laccases, of which eight were predicted to be secreted. Molecular docking predicted substrate binding between − 3.2 to -5.1 ΔG (Table 1), with CEI66207 demonstrating the most favourable binding despite being characterised as a peptidoglycan editing factor (*pgeF*). BLASTp indicated a low percentage identity (29.48%) but high coverage (97%) and a desirable expect value (3.38e-22), akin to that of MCO1. Furthermore, the presence of a signal peptide suggests CEI66207 may contribute toward *F. venenatum* utilisation of plastic hydrocarbons [28] following secretion. Genome mining of a *Salipaludibacillus* species, *S. agaradhaerens*, and novel *Stenotrophomonas* sp. also identified two non-secreted candidate laccases.

**Table 1:** *Fusarium* *venenatum* laccase molecular docking

**Laccases (GenBank Accession)**	**Δ Gibbs free energy (kcal mol**^**-1**^**)**
CEI39947	-3.9
CEI40379	-3.2
CEI41409	-3.7
CEI62062	-3.7
CEI62063	-4.2
CEI63741	-4.2
CEI66016	-4.2
**CEI66207**	**-5.1**
CEI66209	-4.9
CEI67340	-3.8
CEI67690	-4.3
CEI69051	-4.1
CEI70801	-3.6

Comparing the predicted dodecane-binding of 13 uncharacterised, putative laccases encoded by *F. venenatum*. Values represent a single replicate. The laccase selected as a candidate is emboldened

### Selection of candidate CBM domains

**Fig. 1:**
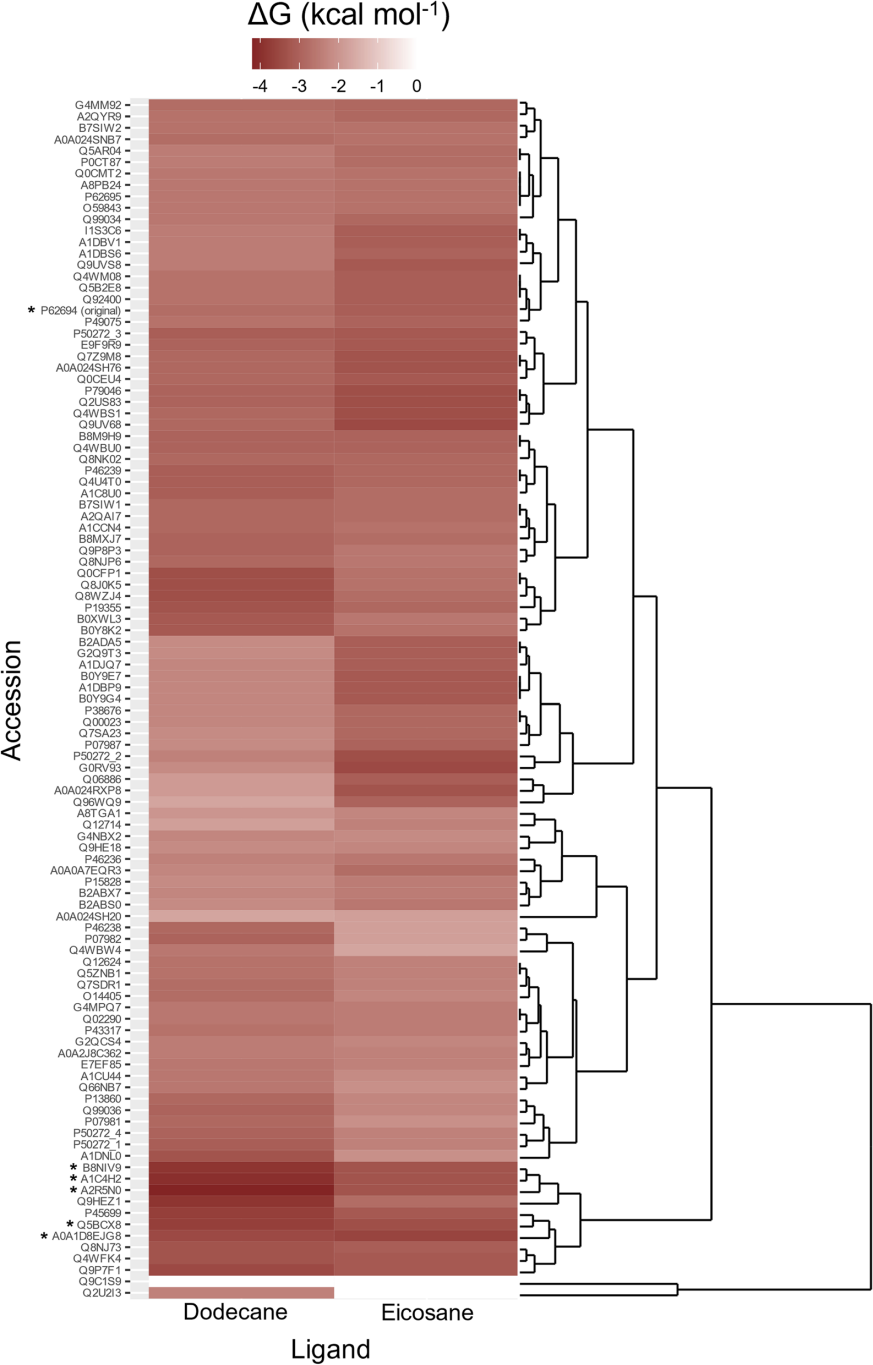
CBM1 domain candidate screening. Molecular docking of 105 CBM1 domains and both dodecane and eicosane ligands. Values were obtained from single replicates. Lower ΔG (kcal mol^− 1^) reflects greater binding affinity. The dendrogram and heatmap were created using ggplot and ggdendro, respectively, in R. *CBM1 domains selected for further analysis

Large-scale molecular docking was performed to screen candidate CBM family 1 domains for consequent generation of laccase chimeras. The CBM family was chosen based on the reported binding with PET [28], which was therefore postulated to bind polyethylene. Both dodecane (12 C) and eicosane (20 C) ligands were used in molecular docking analyses to represent differential hydrocarbon chain lengths and one hundred and five CBM1 domains, including the CBM1 utilised by Dai et al. [28] for PET engineering, were examined. Predicted binding affinities ranged from ΔG -3.6 to 0 (Fig. 1) and five CBM1 domains with the lowest average ΔG for both dodecane and eicosane were taken forward (Uniprot accessions): A2R5N0, A1C4H2, B8NIV9, Q5BCX8, and A0A1D8EJG8. The CBM1 of *T. reesei* (P62694) was retained as a comparator [28], although preliminary docking reported a higher average binding affinity of -2.95 ΔG (Fig. 1).

All five CBM1 candidates were derived from fungi, primarily *Aspergillus* sp. (A2R5N0, A1C4H2, and B8NIV9, Q5BCX8) and *Sodiomyces alcalophilum* (A0A1D8EJG8). The former was identified as putative endo-β-1,4-glucanase D enzymes whilst the latter as a 4-O-methyl-glucuronyl methylesterase. Notably, eicosane reported a lower predicted ΔG than dodecane, except for A0A1D8EJG8.

### Preliminary molecular docking

Three-dimensional models of the protein-ligand interactions were visualised within discovery studio (DS) to examine CBM binding site residues. Of 25 amino acid (AA) residues observed across the five candidates, 16 (64%) were aromatic (tryptophan, tyrosine, or phenylalanine) whilst a further six were aliphatic (leucine, isoleucine, or valine). Therefore, hydrophobic AAs constituted a significant proportion of the predicted CBM binding residues. A CBM2 domain of *Sorangium cellulosum* (GenBank Accession: SOCE26_003050) was included within consequent bioinformatic analyses, with its native linker, to explore differential PE binding of CBM types.

**Fig. 2:**
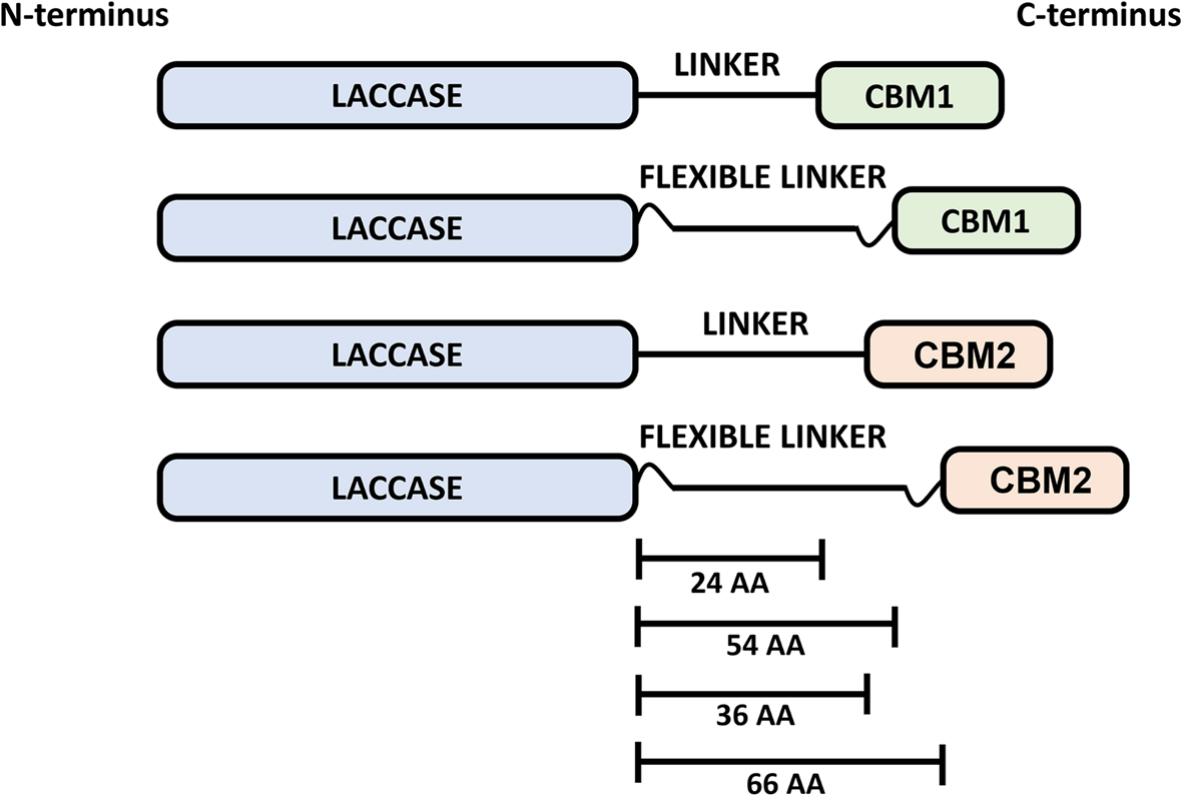
Schematic chimera constructs. Representation of chimera construct components. Scale bars demonstrate the length of linkers, both with and without flexible hinges

Chimeric enzymes were constructed using one of the four candidate laccases, the linker of *T. reesei* [28], and one of the five candidate CBM domain (Fig. 2). Despite native CBM2 N-terminal positioning, consensus C-terminal positioning was chosen to facilitate comparison to the seminal *T. reesei* CBM utilised by Dai et al. [39].

**Fig. 3:**
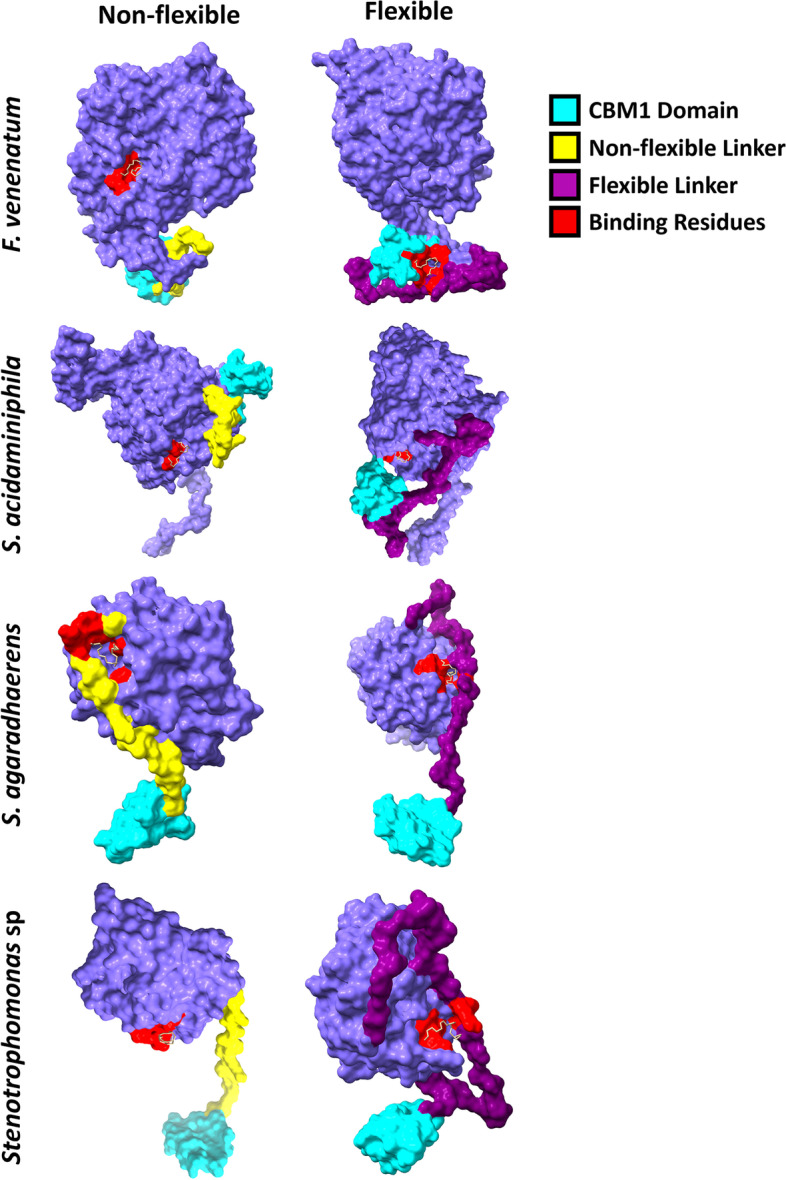
Three-dimensional visualisation of protein-ligand binding. Molecular docking protein-ligand poses were visualised and annotated within ChimeraX. Binding residues were identified within Discovery Studio. Non-flexible constructs were compared to flexible chimeras to visualise differences in ligand binding following the addition of GGGGS(x3) hinges. Laccases derive from *Fusarium venenatum*, *Stenotrophomonas acidaminiphila*, *Salipaludibacillus agaradhaerens*, and a novel *Stenotrophomonas* sp. See Additional file 2 for laccase sequence information. Displayed models are representative of triplicate docking analyses

Molecular docking was performed for each of the chimeric variants and the protein-ligand binding interactions visualised (Fig. 3). It was observed that many interactions were predicted to lack direct CBM involvement. Therefore, GGGGS(x3) hinges were incorporated into both ends of the linker to generate “flexible” chimeric enzymes (Fig. 2). This increased linker length by 30 AAs. The linker and/or CBM domain of these flexible constructs were predicted to have closer associations with the binding site/residues within molecular docking simulations, with direct ligand binding predicted for flexible *F. venenatum* and *S. acidaminiphila* chimeras (Fig. 3). To investigate further the impacts of both GGGGS(x3) hinges and CBM domains, correlation and analysis of variance (ANOVA) *post hoc* testing were performed on both quantitative binding and qualitative enzyme properties, respectively.

### Relationships between quantitative enzyme variables

To explore potential relationships between quantitative enzyme variables, correlation analyses were conducted, and dissociation constants (Kd, µM) were derived from molecular docking ΔG output and used to interpret substrate binding affinities. Specifically, Kd reflected the concentration at which half the ligand-binding sites of a protein were saturated, with lower values representing stronger binding. Catalytic constant (k_cat_, s^− 1^) was predicted using the Deep-Learning k_cat_ (DLKcat) algorithm [40]. In addition, other variables considered were enzyme stability, hydropathicity, the binding site surface area, and distance between ligand and CBM domain in simulated protein-ligand interactions of chimeric constructs. Stability was reported as an instability index, with values > 40 suggesting instability [41]. Hydropathicity was measured by aliphatic index [42] and Grand Average of Hydropathy (GRAVY) [43], with larger values in both variables reflecting greater predicted hydrophobicity.

**Fig. 4:**
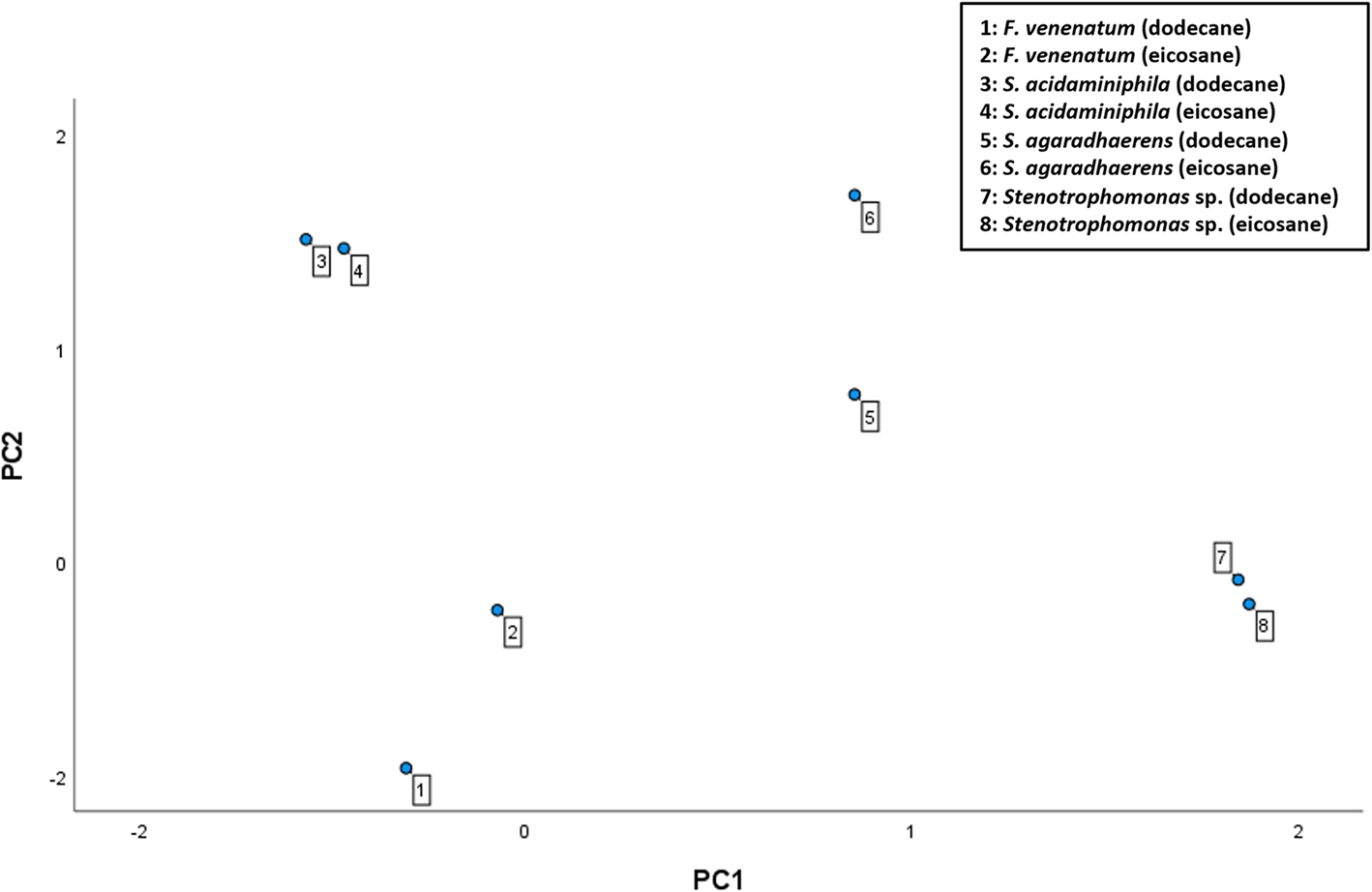
Separation of samples by unsupervised Principal Component Analysis. Principal component analysis was performed within SPSS. Input data were grouped by laccase type into rows and dependent variables as columns. Two principal components were used to discern variance between laccases, grouping similar samples together. Numerical data labels reflect different enzyme variants, reflected within the above legend

Preliminary unsupervised principal component analysis (PCA) was performed to explore the potential influence of different laccases on the dependent variables measured. Using Wild Type (WT) samples, PCA separated samples into their cognate laccases, independent of the ligand (Fig. 4). Laccases of *F. venenatum* (1 & 2) and *S. agaradhaerens* (5 & 6) demonstrated greater ligand-dependent separation from the principal component two (PC2) classifier, however, which was most strongly correlated to k_cat_ value (r^2^ = 0.558). PC1, in contrast, was correlated strongly with Kd, instability, and hydropathicity variables. Following PCA analysis, bivariate correlation between enzyme parameters was investigated for all laccases independently along with the total correlation of all protein variants.

**Fig. 5:**
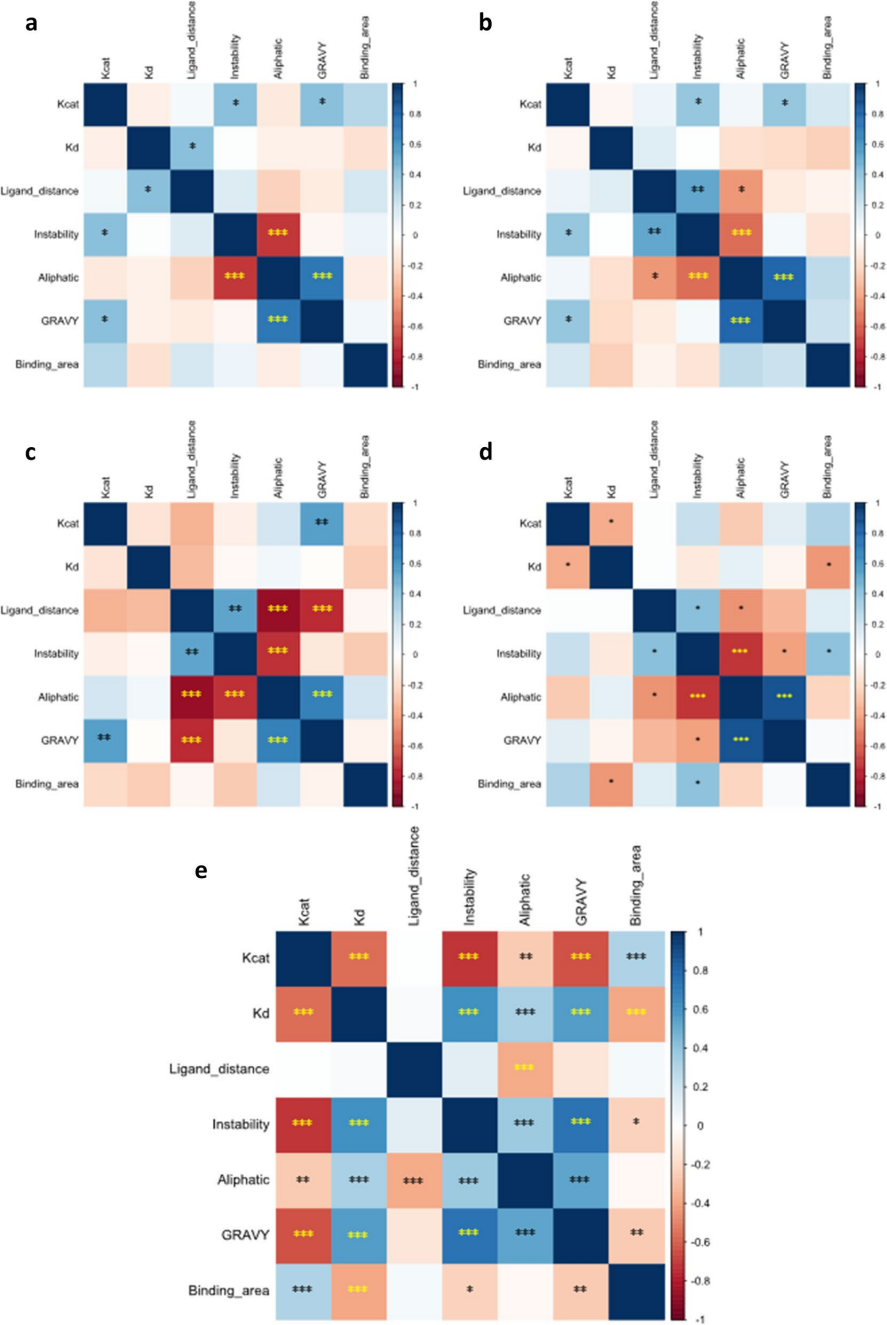
Correlation between enzyme dependent variables. Correlation analysis was performed on *F. venenatum *(**a**), *S. acidaminiphila *(**b**), *S. agaradhaerens *(**c**), *Stenotrophomonas* sp. (**d**), and Total (**e**) samples. Statistical analysis and correlation plotting was performed using corrplot package in R. Colours reflect r values. *p* < 0.05(*); *p* < 0.01(**); *p* < 0.001(***)

Total correlation was performed using all 120 samples (Fig. 5). Highly significant associations were reported for both k_cat_ and Kd, demonstrating a moderative negative association (*r* = -0.559) of very high statistical significance (*p* < 0.001) between one another, with lower Kd values observed within those with higher k_cat_ measurements. k_cat_ also had a strong negative association with instability index (*r* = -0.712, *p* < 0.001), and a moderate negative association with GRAVY (*r* = -0.636, *p* < 0.001). A weak positive association was observed between k_cat_ and binding site surface area (*r* = 0.3, *p* < 0.001) and aliphatic index (*r* = -0.251, *p* < 0.001), with negligible association with the distance between ligand and CBM (*r* = 0.009, *p* = 0.918). In other hand, Kd reported a moderate positive correlation with instability index (*r* = 0.608, *p* < 0.001) and GRAVY (*r* = 0.558, *p* < 0.001) with further weak positive (*r* = 0.327, *p* < 0.001) and negative (*r* = -0.382, p < 0.001) associations with aliphatic index and binding site surface area, respectively. Like k_cat_, no associations were observed between Kd and the distance between ligand and CBM (*r* = 0.023, *p* = 0.8). However, this variable did observe weak negative correlation with aliphatic index (*r* = -0.374) of very high statistical significance (*p* < 0.001). In contrast, no further associations were observed with instability (*r* = 0.126, *p* = 0.169), GRAVY (*r* = -0.136, *p* = 0.137), or binding site surface area (*r* = 0.041, *p* = 0.656). Instability index correlated with all variables except for the distance between ligand and CBM. There were moderate positive (*r* = 0.368, *p* < 0.001) and negative (*r* = -0.222, *p* = 0.015) correlation with aliphatic index and binding site surface area, respectively. A strong positive correlation was also observed between instability and GRAVY (*r* = 0.733, *p* < 0.001). Aliphatic index associated with all variables with the exception of binding site surface area (*r* = -0.32, *p* = 0.725), including a moderate positive association with GRAVY (*r* = 0.528, *p* < 0.001). Similarly, GRAVY correlated with all other variables except for the distance between ligand and CBM, with a weak negative association with binding site surface area (*r* = -0.254, *p* = 0.005).

Figure 5 suggested differential, laccase-dependent, associations (Fig. 5), in agreement with Fig. 4. Notably, fewer significant associations were observed although lower sample sizes were also used for the bivariate correlation analyses (*n* = 30). *A priori* and *post hoc* power analyses using k_cat_ and Kd correlation suggested a sample size of 31 was required to detect the moderate effect size between these variables (*r* = -0.559), with an approximate power value of 0.945 obtained with the individual laccase sample size. Further, sensitivity power analysis using a power value of 0.8 and alpha value of 0.5 suggested laccase-dependent correlation analyses can detect significant associations with a moderate effect size (*r* = 0.468), whilst a sample size of 120 increased the sensitivity of finding statistically significant correlations to those of weak effect size (*r* = 0.249).

Most connections were observed, as a general consensus, between the different laccases and the total correlation, including associations between k_cat_ and Kd (negative); k_cat_ and the distance between ligand and CBM (negligible); k_cat_ and aliphatic index (negligible/weak negative); k_cat_ and binding site surface area (positive); Kd and the distance between ligand and CBM (negligible); Kd and aliphatic index (negligible); Kd and binding site surface area (negative); the distance between ligand and CBM and aliphatic index (negative), GRAVY (negligible), and binding site surface area (negligible); instability and binding site surface area (negative); aliphatic index and binding site surface area (negligible); and GRAVY and binding site surface area (negligible) (Fig. 5). In contrast, several variable correlations were highly distinct between different laccases and compared to the total correlation reported. For example, k_cat_ and instability index reported strong negative total correlation (*r* = -0.712, *p* < 0.001) despite general weak positive associations for independent laccases. Similar observations were made for k_cat_ and GRAVY. Aliphatic index and GRAVY also reported differential associations with total Kd. Further, despite moderate positive associations between instability index and the distance between ligand and CBM in *S. acidaminiphila* (*r* = 0.517, *p* = 0.003), *S. agaradhaerens* (*r* = 0.525, *p* = 0.003), and *Stenotrophomonas* sp. (*r* = 0.417, *p* = 0.022) laccases, the total correlation reported was negligible (*r* = 0.126, *p* = 0.169). A similar trend was observed for instability index and GRAVY correlation. Interestingly, aliphatic index and GRAVY correlations were highly differential amongst different laccases, with strong positive associations reported for both *Stenotrophomonas* species (*r* = 0.796–0.867, *p* < 0.001) but strong and moderate negative associations reported for *F. venenatum* (*r* = -0.714, *p* < 0.001) and *S. agaradhaerens* (*r* = 0.671, *p* < 0.001), respectively. A consensus moderate positive correlation (*r* = 0.528, *p* < 0.001) was reported for total samples. To explore the importance of enzyme properties on these enzyme kinetic variables, ANOVA *post hoc* analyses were performed.

### Influence of protein properties on k_cat_

To better understand the impact of different protein characteristics on enzyme catalysis, k_cat_ was predicted with simulated binding to both dodecane and eicosane. These values were pooled for analyses.

**Fig. 6:**
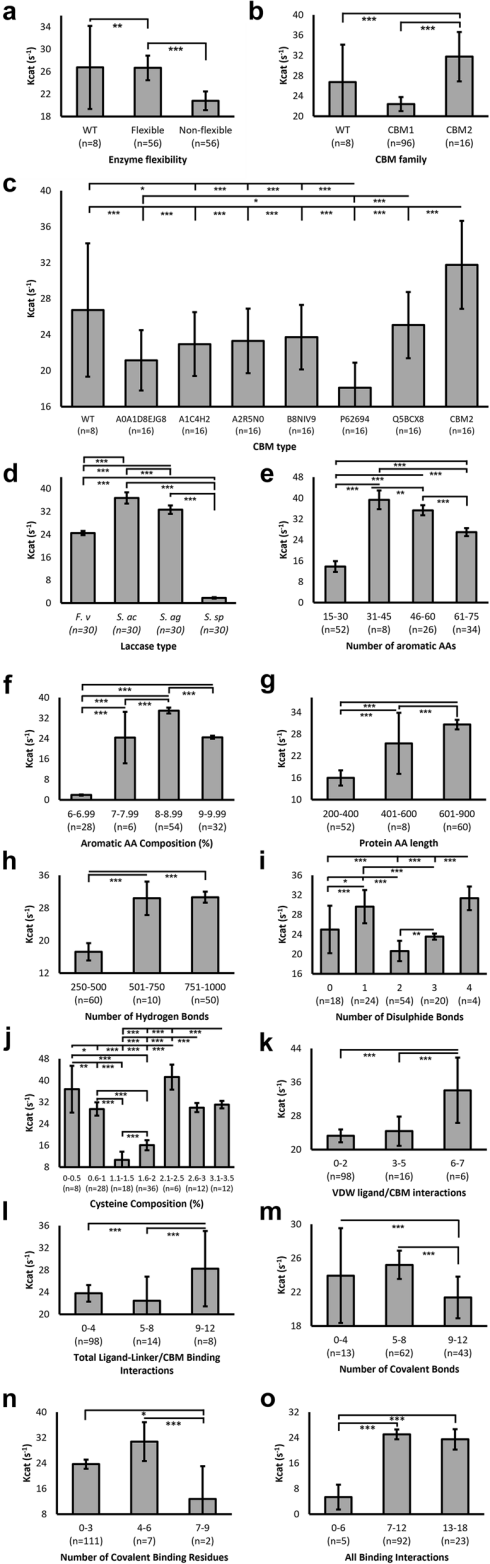
The effects of protein properties on catalytic activity. Catalytic activity was predicted using DLKCat algorithm [40] and reported as a turnover number (s− 1). Averages were plotted, with sample sizes (n) displayed underneath each bar. Error bars represent SEM. Statistical significance calculations were performed as post hoc Tukey tests following univariate ANOVA. *p* < 0.05(*); *p* < 0.01(**); *p* < 0.001(***)

Non-flexible chimeras reported significantly reduced k_cat_ compared to WT enzymes, despite a small sample size in the latter (*n* = 8, Fig. 6a). k_cat_ was also significantly higher in flexible constructs, at a similar level to that observed in WT variants. CBM1 chimeras reported significantly reduced k_cat_ compared to WT enzymes, whilst CBM2 displayed significantly higher predicted k_cat_ compared to both CBM1 (1.41-fold) and WT (1.18-fold) (Fig. 6b). It was also highly dependent on CBM type (Fig. 6c), where CBM2 yielded significantly higher k_cat_ (31.746 s^− 1^) than all CBM1 variants. Interestingly, all CBM1 variants reported non-significantly reduced k_cat_ compared to WT. However, note the latter comprised a small sample size (*n* = 8). Chimeras containing the CBM1 utilised by Dai et al. [28] reported the lowest predicted k_cat_ value (18.095 s^− 1^), significantly lower than all other CBM1 and CBM2 variants. k_cat_ varied significantly between all laccase types (Fig. 6d), particularly between the two *Stenotrophomonas* laccases, with *S. acidaminiphila* observing the highest activity of all laccases (36.744 s^− 1^) whilst *Stenotrophomonas* sp. reported a 20.4-fold lower value (1.802 s^− 1^). Both the number of aromatic AAs (Fig. 6e) and the aromatic AA composition (Fig. 6f) demonstrated significantly differential k_cat_ values. Generally, more aromatic AAs and thus a higher composition reported higher k_cat_, although this peaked at around 8-8.99% aromatic AA composition followed by a reduction as the number of aromatic AA residues increased. Similarly, longer proteins reported significantly higher values (Fig. 6g), with a linear trend between protein length and k_cat_ values. Those between 601 and 900 AAs reported an average 1.2-fold and 1.9-fold greater k_cat_ than those between 401 and 600 and 200–400, respectively. The number of hydrogen bonds formed within each sample also significantly differed in predicted k_cat_ (Fig. 6h), with those forming more hydrogen bonds (between 501 and 1000) reporting significantly higher values than proteins with fewer hydrogen bonds between 250 and 500 (1.77-fold). Disulphide bonds formed within proteins, whilst differing significantly amongst one another, did not reflect a linear k_cat_ relationship, with smaller samples sizes across a larger number of groups (Fig. 6i). Nevertheless, statistically significant improvements in k_cat_ were observed when < 2 or ≥ 4 disulphide bonds were formed, with those forming 2 or 3 reporting poorer activity. Surprisingly, this bimodal distribution was similar conserved within cysteine composition of proteins (Fig. 6j), with high k_cat_ values reported for proteins with between 0 and 1 and 2.1–3.5% cysteine composition, whilst those with 1.1-2% reported significantly lower activity. A linear increase in k_cat_ was predicted with increasing number of Van der Waals (VDW) interactions between the ligand and CBM domain (Fig. 6k), albeit with a low sample size for > 6 VDW bonds formed (*n* = 6). This phenomenon was also observed in the number of all bonds formed between ligand and linker/CBM, with those forming > 9 reporting significantly higher k_cat_ (28.24 s^− 1^) compared to those with 5–8 (22.429 s^− 1^) and 0–4 (23.781 s^− 1^) bonds formed (Fig. 6l). Akin to trends observed in aromatic AA number/composition, the number of covalent bonds formed (Fig. 6m) and residues forming covalent bonds (Fig. 6n) increased concurrently with k_cat_ however yielded reduced turnover numbers above a threshold. Specifically, 5–8 covalent bonds formed between protein and ligand demonstrated non-significantly higher k_cat_ than those with 0–4 bonds but significantly higher also than those with 9–12 bonds. This was corroborated within the number of residues forming covalent bonds also, with 4–6 covalent residues reporting significantly higher k_cat_ than those forming 0–3 and 7–9 (Fig. 6n). It is important to state that the majority (*n* = 111) of samples observed between 0 and 3 covalent binding residues within the protein-ligand interaction, whereas those forming > 3 were less frequent (*n* = 9). When considering total binding interactions formed between protein and ligand (VDW and covalent), greater binding prevalence yielded significantly increased k_cat_ compared to those forming < 7 total bonds (Fig. 6o). There were no statistically significant improvements in k_cat_ reported when protein-ligand interactions consisted of > 7 bonds.

### Influence of protein properties on kd

Binding affinity was assessed using Kd, with more infrequent statistical significance and larger Standard Error of the Mean (SEM) attained compared to k_cat_.

**Fig. 7:**
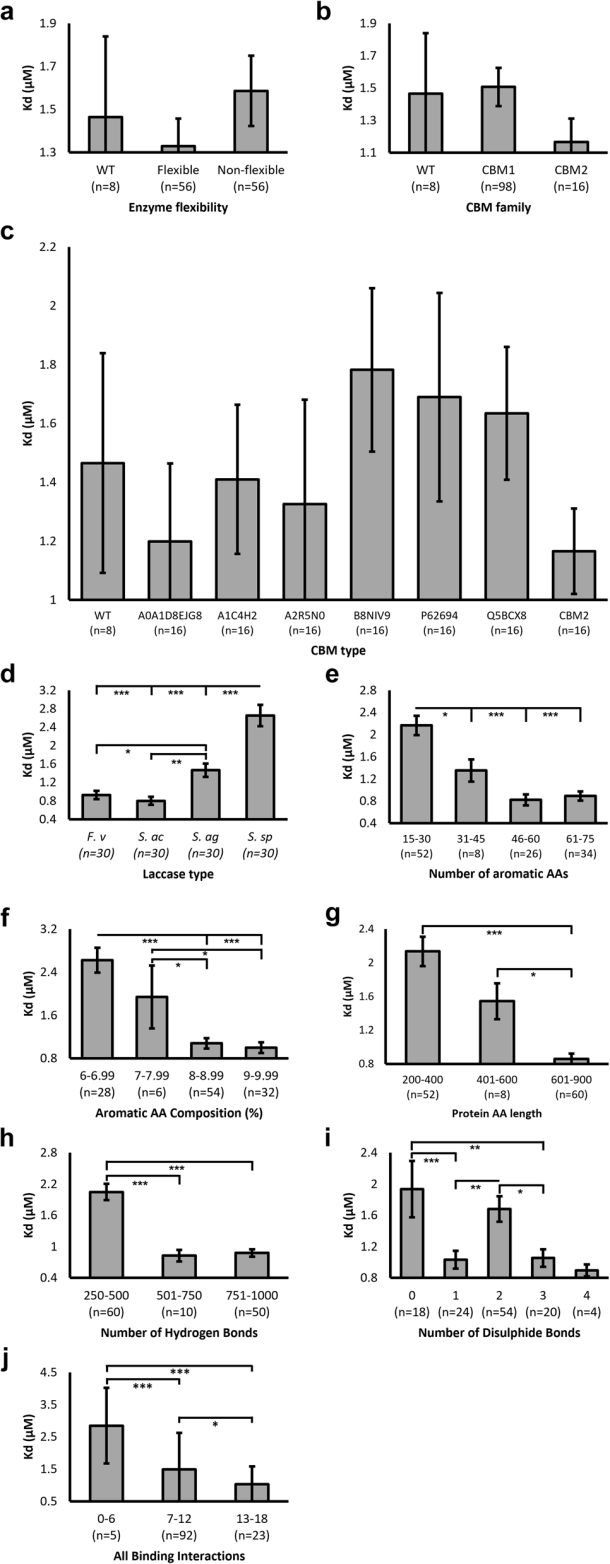
The effects of protein properties on binding affinity. Binding affinities were expressed as Kd (µM), derived from Gibbs Free Energy of molecular docking simulations. A temperature of 298 K was applied. Averages were plotted from triplicate docking analyses, with group sample sizes (n) displayed underneath each bar. Error bars represent SEM. Statistical significance calculations were performed as *post hoc* Tukey tests following univariate ANOVA. *p* < 0.05(*); *p* < 0.01(**); *p* < 0.001(***)

As with k_cat_, non-flexible chimeras yielded detrimental effects, with 1.08-fold higher Kd observed compared to WT (Fig. 7a). Further, flexible Kd was lower and thus improved compared to both WT and non-flexible variants, akin to that observed for Kcat. CBM2 chimeras possessed favourable ligand binding affinity (1.165 µM) compared to both WT (1.465 µM) and CBM1 (1.506 µM) (Fig. 7b). When examined in greater detail, A0A1D8EJG8 reported the most favourable Kd of CBM1 domains, like that of CBM2 **(**Fig. 7c). The CBM1 of *T. reesei* utilised by Dai et al. [28] reported the second poorest Kd value (1.689 µM). Surprisingly, akin to that observed in k_cat_, several CBM1 chimeras reported poorer Kd compared to the WT enzyme. The different laccases reported significantly differential Kd values (Fig. 7d), with relatively low SEM and evenly distributed sample size per group (*n* = 30). *Stenotrophomonas* sp. demonstrated the least favourable Kd (2.649 µM), 3.3-fold higher than the most favourable predicted binding reported in *S. acidaminiphila* (0.798 µM) (Fig. 7d). The linear improvements in Kd with increasing aromatic AA composition mimicked observations made for k_cat_, with more aromatic AA residues (Fig. 7e) and thus greater aromatic AA composition (Fig. 7f) yielding significantly improved putative binding affinity. This was the same for protein length, with longer proteins > 600 AA reporting significantly lower Kd (0.86 µM) compared to those ≤ 600 AA in length (1.544–2.134 µM) (Fig. 7g). Protein-ligand interactions with > 500 hydrogen bonds reported significantly lower Kd (~ 2.4-fold) than those with fewer hydrogen bonds (Fig. 7h). Similarly, a bimodal distribution was reported for the number of disulphide bonds for Kd (Fig. 7i), with proteins forming 0 or 2 disulphide bonds reporting significantly poorer Kd compared to those with 1, 3, or 4 bonds, although interpretation was limited again by low sample sizes in certain groups. Generally, the more protein-ligand binding interactions formed, the lower the Kd and thus strong binding affinity predicted (Fig. 7j). Most proteins formed between 7 and 12 bonds with the ligand, whilst those forming 13–18 bonds reported significantly improved Kd.

### Influence of protein properties on protein stability

The instability index considers protein length and the presence of particular dipeptides and AA residues shown to correlate with differential in vivo stability [41]. It is suggested that values < 40 are to be considered stable.

**Fig. 8:**
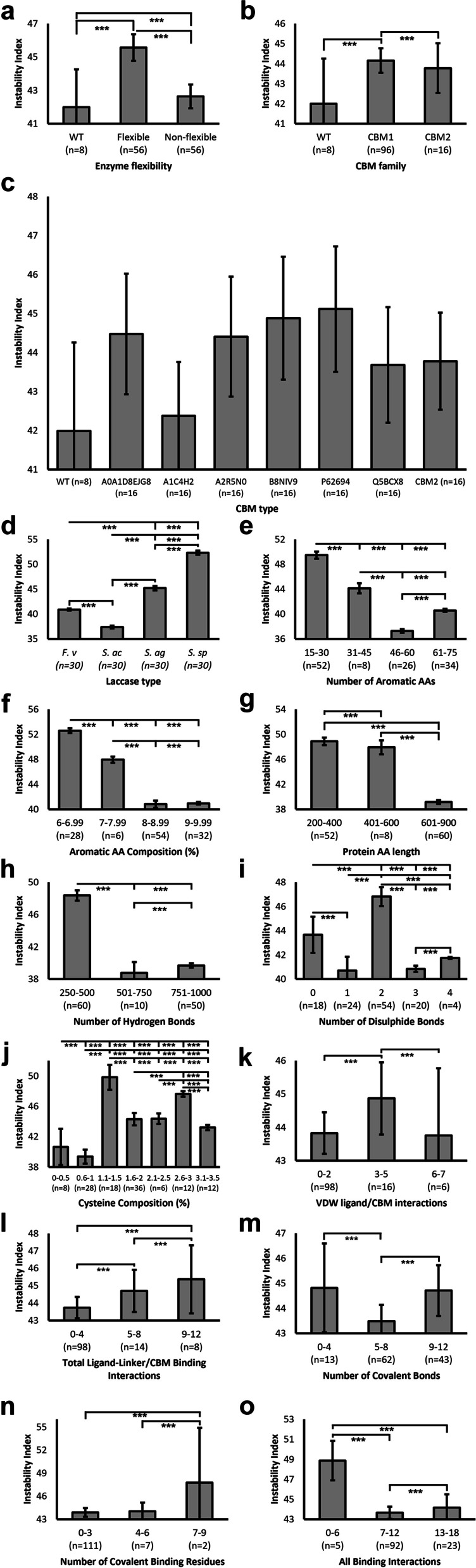
The effect of protein properties on stability. Instability index was predicted through ProtParam, using the formula proposed by Guruprasad et al. [41]. Averages were plotted, with sample sizes (n) displayed underneath each bar. Error bars represent SEM. Statistical significance calculations were performed as *post hoc* Tukey tests following univariate ANOVA. *p* < 0.05(*); *p* < 0.01(**); *p* < 0.001(***)

Notably, average instability indices were > 40 for most independent variables. For example, the WT, flexible, and non-flexible samples all reported putative instability (Fig. 8a). Flexible constructs reported significantly higher instability than both WT and non-flexible constructs (45.573). This was replicated in Fig. 8b, with both CBM1 and CBM2 chimeras predicted to be significantly less stable than WT variants. When considering each CBM1 domain separately (Fig. 8c), A1C4H2 displayed the most favourable predicted stability yet was still > 40 and above WT. Surprisingly, the A0A1D8EJG8 and CBM2 domains demonstrating the most favourable k_cat_ and Kd did not demonstrate acceptable stability. Note that the latter reported a moderate positive correlation with instability index whilst the former reported a strong negative association. Of the different laccases, *S. acidaminiphila* enzymes were the only group to report predicted stability (37.41), whilst all other enzymes reported significantly higher instability (Fig. 8d). In concurrence with Kd and k_cat_ observations thus far, *Stenotrophomonas* sp. laccases reported the highest predicted instability (52.321). Generally, increased stability was observed in proteins with greater numbers of aromatic AAs (Fig. 8e) and thus composition (Fig. 8f). However, akin to observations made for Kd, > 60 aromatic AAs reported significantly higher instability than those between 46 and 60, although still significantly lower than that reported for < 45 (Fig. 8e). Expectedly, owing to the methodology of instability index prediction, protein length predicted linear improvements in protein stability within longer proteins (Fig. 8g), with those > 600 AAs in length reporting significantly improved stability prediction. Similarly, increased number of intra-protein hydrogen bonds significantly improved putative stability (Fig. 8h). As observed for k_cat_ and Kd, the number of disulphide bonds (Fig. 8i) and cysteine composition (Fig. 8j) significantly affected protein instability non-linearly. Those forming 0 or 2 disulphide bonds reported significantly poorer protein stability, whereas those forming 1, 3, or 4 disulphide bonds reported significantly lower instability index. Expectedly, higher numbers of intra-protein bonds formed significantly improved putative stability (Fig. 8o). It is noteworthy that this was reflected within VDW-ligand interactions (Fig. 8k) but not, however, in the number of total ligand-linker/CBM interactions (Fig. 8l) or covalent bonds (Fig. 8m, n), although it should be stated that underrepresentation within higher bond groups yielded low sample sizes and concurrently higher SEM.

### Influence of protein properties on the distance between ligand and CBM domain

The distance between ligand and CBM in protein-ligand docking poses was measured and compared between independent protein properties, examining their potential influence on ligand binding.

**Fig. 9:**
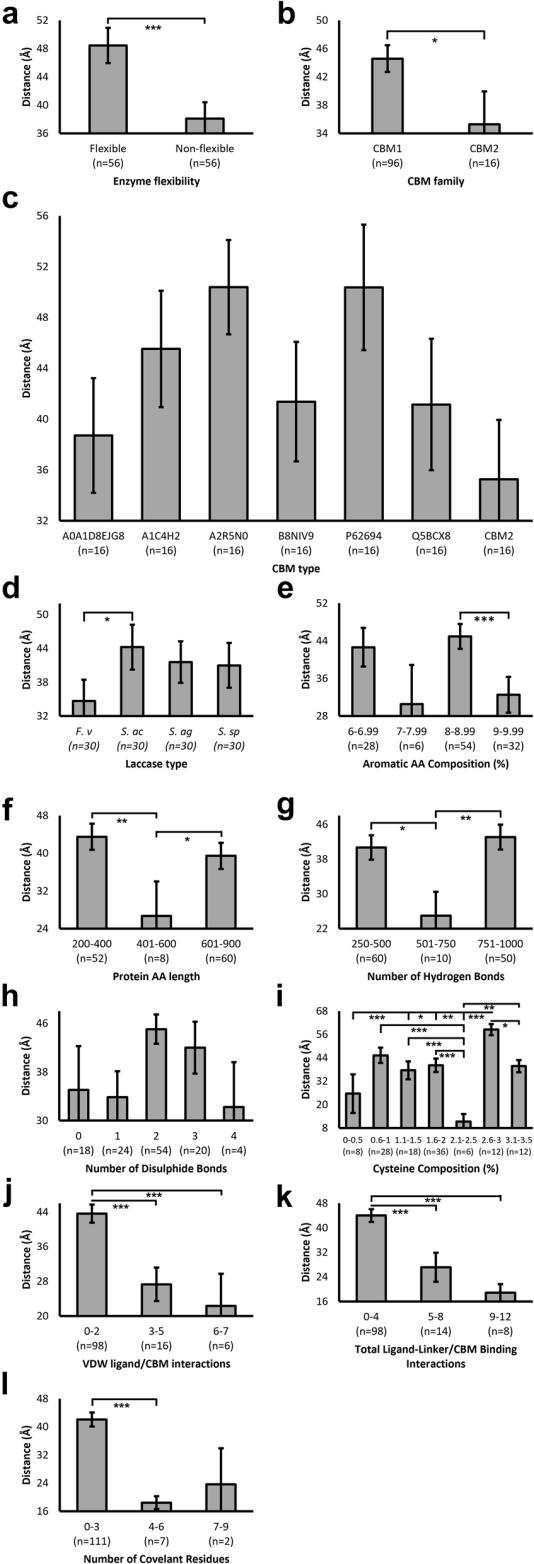
The effect of protein properties on the distance between ligand and CBM domain. CBM-ligand distance was calculated within Pymol. The average distance between each ligand carbon atom and the alpha carbon of the first CBM AA residue were displayed. Averages were plotted, with sample sizes (n) displayed underneath each bar. Error bars represent SEM. Statistical significance calculations were performed as *post hoc* Tukey tests following univariate ANOVA. *p* < 0.05(*); *p* < 0.01(**); *p* < 0.001(***)

Unexpectedly, a significantly larger distance between ligand and CBM was reported for flexible compared to non-flexible chimeras (Fig. 9a). Despite this, CBM2 chimeras demonstrated significantly lower (1.26-fold) binding distance than CBM1 (Fig. 9b). No significant differences were observed between CBM1 types (Fig. 9c). However, large SEM values for each group and relatively small sample sizes (*n* = 16), or an extraneous parameter, may influence this. An example is laccase type, in which *F. venenatum* reported significantly reduced binding distance compared to that of *S. acidaminiphila* (Fig. 9d). Whilst the number of aromatic AA residues did not significantly impact ligand binding distance, the aromatic AA composition of proteins reported significantly reduced binding distance in those with 9-9.99% compared to 8-8.99% (Fig. 9e). Despite similarly reduced binding distance reported in those with 7-7.99%, low sample size and thus high SEM influence statistical significance and also potentially the data average recorded. This phenomenon was repeated with protein length (Fig. 9f), the number of hydrogen bonds (Fig. 9g), number of disulphide bonds (Fig. 9h), and cysteine composition (Fig. 9i), with the former lacking statistically significant findings (Fig. 9h), whilst adequate sample size of ~ 30 previously discerned was only satisfied by a single group. In contrast, highly significant differences were reported despite low sample sizes for protein length, hydrogen bond number, and cysteine composition, with those containing 2.1–2.5% cysteines reporting significantly lower average ligand binding distance (Fig. 9i). Generally, akin to observations made for k_cat_, Kd, and instability, greater number of protein-ligand bonds significantly decreased the putative binding distance (Fig. 9j-l), although the majority of samples formed < 4 VDW (Fig. 9j, *n* = 98), covalent (Fig. 9l, *n* = 111), or total bonds (Fig. 9k, *n* = 98) bonds, limiting interpretation of these results.

## Discussion

### Screening laccase and CBM candidates

Candidate identification is a necessity before consequent engineering. Here, a genome mining approach permitted high-throughput, rapid identification of laccases/MCOs, focusing on genera commonly isolated from PE waste and those being researched by our group. Utilisation of raw RNA-sequencing data enabled the extraction of a laccase proposed to mediate the PE biodegradation of *R. opacus* [27], for use as a genome mining probe. This signifies the importance of providing data, particularly deriving from large datasets, for research progression. Indeed, from this, two putative MCOs of *S. acidaminiphila* were obtained. Low identity but high query coverage suggested conserved function but differential structure(35), indicating orthologous proteins with potentially distinct functional properties, including substrate specificity and catalytic efficiency. Similar criteria were satisfied when identifying the putative MCO of *S. agaradhaerens*, suggesting misclassification as a peptidoglycan editing factor and thus requiring re-examination as a likely oxidase, supported by a low expect value. A pilot molecular docking study revealed improved binding affinity predicted for both MCOs of *S. acidaminiphila* compared to that of Santacruz-Juarez [38], identifying MCO1 as a desirable candidate for further analysis. High variability is an important limitation of molecular docking, however, suggesting the single replicate performed may not adequately represent the capabilities of these enzymes. Therefore, it is of interest, particularly following the enzyme kinetics observed for MCO1 chimeras, to examine the potential of MCO2. This is applicable also to the laccases of *F. venenatum*, which displayed even greater predicted binding. The predicted extracellular localisation of *F. venenatum* and *S. acidaminiphila* candidate laccases supports their native role as exoenzymes. Despite predicted non-secretion of the novel *Stenotrophomonas* sp. laccase, putative intracellular processing of small fragment PE can be proposed due to the presence of a co-localised ABC transporter. However, this would need experimental validation. Furthermore, recombinant bioengineering of candidate laccases negates the importance of native localisation, focusing rather on activity and substrate preference. The CBM screening approach used in this study is a high-throughput method, adaptable to different substrates, providing rapid and thorough identification of candidates without significant experimental restraints. It is therefore a highly useful workflow for guiding directed evolution. For example, the five CBM1 domains selected from large-scale screening (Fig. 1) reported significantly more favourable catalytic activity than the CBM1 used successfully by Dai et al. [28] for PET targeting. This highlights both the usefulness of CBM screening and differential binding affinities between CBM of the same family. All five CBM1 candidates derived from fungal species. Unsurprisingly, three of these belong to *Aspergillus* sp., a genus frequently isolated from PE-contaminated sites [17, 44–46]. Whilst this suggests fungal CBMs are superior to those deriving from bacteria, the accessibility of large-scale screening negates these assumptions, allowing potential outliers to emerge from bacterial sequences. Interestingly, four of five CBM1s derived from putative endo-β1,4-glucanases. It could be postulated that PE and beta-glucans therefore share similar physicochemical properties explaining apparent substrate-specificity crossover. Akin to the crystallinity and hydrophobicity of cellulose, potential similarities between PE and beta-glucans are worth exploring.

### *k*_*cat*_ prediction reliability

Along with dodecane and eicosane as PE representatives, guaiacol was chosen as a substrate for in silico k_cat_ predictions. A small pilot study was performed, using 11 laccases deriving from the BRENDA database [47], to examine laccase-specific accuracy of the k_cat_ algorithm (see Additional file 1 for data). Compared to a high correlation (*r* = 0.87) between predicted and experimental k_cat_ values reported by the authors [40], a negligible correlation of 0.069 was obtained in the pilot study. Despite this, the large sample size of 7,822 sequences used to test the algorithm [40] supported its use within this study, however only as a predictor, to be interpreted with care.

### Binding affinity and catalytic activity are influenced by protein stability and binding site size

In the interest of discerning broad associations between enzyme properties and PE oxidation, total correlation analyses were favourable to interpret, with the aim of elucidating trends applicable to general laccase optimisation. As such, Kd and k_cat_ association suggested increased binding affinity concurrently improved catalytic activity. This was potentiated within more stable enzymes. Engineering laccases to increase PE binding will therefore improve oxidation capabilities, bearing major significance within industrial-scale applications of this technology to improve rate-limiting turnover rates. Correlation between the binding site surface area and catalytic activity (Fig. 5e) of a protein suggests larger enzymes may be more efficient oxidases, with increased volume encouraging substrate/cofactor binding. As hydrophobic interactions have been previously attributed to facilitate CBM-PE binding, negative association between hydrophobicity and both binding affinity and consequent catalytic activity (Fig. 5e) was unexpected. Differential enzyme kinetics between laccases, as determined by both *post hoc* (Figs. 6, 7, 8 and 9) and PCA (Fig. 4) analyses, may explain these findings. Indeed, chimera hydrophobicity is largely dependent on its laccase, constituting most of the protein, thus influencing this measured association to a greater extent than the CBM domain. Similarly, the distance between ligand and CBM was hypothesized to reflect substrate binding affinity. However, greater ligand-CBM distances within flexible constructs directly contrasts their improved binding affinities. Supported by negligible associations to both k_cat_ and Kd, it is apparent that this variable is poorly representative of binding. This may be attributable also to the method of calculation, with the distance between ligand and first AA CBM residue measured. It may be more appropriate, therefore, to examine the distance between ligand and C-terminal CBM AA, or to those of predicted binding residues.

### CBM2 domains improve enzyme kinetics over CBM1 domains

All CBM1 chimeras reported reduced catalytic activity (Fig. 6b) and binding affinity (Fig. 7b) compared to WT enzymes. This is supported by previous literature, with Brunecky et al. [48] reporting similar findings whilst Dai et al. [28] suggested chimeric enzymes can detriment enzyme activity. In contrast, CBM2 chimeras reported significantly improved PE binding and catalysis, compared to both WT and CBM1 constructs. This was highly unexpected, with previous research suggesting CBM1 domains facilitate stronger ligand-protein binding [38, 49–51], with contrasting outcomes comparing the two CBM families [24, 48]. However, these mostly concern cellulose binding, not PE. These observations support the highly variable, substrate-dependent, PE binding affinity postulated between CBM families [52], strongly suggesting CBM2 domains are favourable for PE binding and thus exoenzyme engineering. Despite reporting the most favourable enzyme kinetics, CBM2 and A0A1D8EJG8 CBM1 chimeras reported poor stability. This is likely a manifestation of the parameters for calculating instability index. Specifically, the high prevalence of unstable AA residues reported by Guruprasad et al. [41]: methionine, glutamine, proline, glutamic acid, and serine. The composition of these residues explains this phenomenon. The increasing predicted instability is linear with the composition of these residues: WT and A1C4H2 (23.9%); A0A1D8EJG8 (24.3%); CBM2 flexible (25.3%) and non-flexible (25%) (Fig. 8c). A caveat to this observation is the *Stenotrophomonas* sp. laccase reporting 21.9% unstable AA composition yet the greatest instability. This can be explained by the consideration of protein length in instability index calculations [41], with *Stenotrophomonas* sp. laccase being the smallest enzyme. It is unclear as to whether instability indices are therefore accurate, particularly as type A CBMs have been reported to enhance enzyme stability by ~ 40% [25].

### PE binding and oxidation is laccase-dependent

Akin to the differential hydrophobicity between laccases, both catalytic activity and binding affinity are enzyme-dependent, reinforcing the importance of screening exoenzyme candidates. This is supported by sample grouping observed by unsupervised PCA, with vertical separation of *F. venenatum* and *S. agaradhaerens* reflecting ligand-dependent variation in catalytic activity (Fig. 4). It can therefore be postulated that PE substrate length will affect oxidation efficiency, with hydrophobicity increasing linearly with hydrocarbon content [53]. As two short-chain hydrocarbons were used in this study, it is expected that hydrophobic interactions between CBM and PE will increase binding affinity in longer substrates. This is of particular importance considering the necessity to oxidise and depolymerase longer hydrocarbons to permit microbial uptake. Whilst clearly demonstrating poorer PE oxidation potential compared to other laccase candidates, the *Stenotrophomonas* sp. laccase is a useful candidate for future directed evolution research owing to its confirmed role in PE utilisation. In contrast, MCO1 of *S. acidaminiphila* reported high catalytic activity and binding affinity for PE, albeit lower than has been previously predicted [38].

### Flexible linkers significantly improve chimeras

Linkers are necessary for normal protein expression [49, 54] and cellulase catalysis, retaining activity even in CBM-truncated mutants [51, 52, 55]. They are therefore significant determinants in the efficacy of chimeric enzymes. GGGGS(x3) hinges has been shown to convey flexibility, particularly due to glycine residues [56], yielding stronger binding interactions [30, 56]; they were therefore hypothesized to improve PE binding. Whilst variable differences were indicated from three-dimensional protein-ligand visualisation (Fig. 3), *post hoc* testing demonstrated the importance of flexible hinges for optimised PE oxidation. Flexible chimeras rescued the reduced catalytic activity and binding affinity of non-flexible constructs (Figs. 6a and 7a). This is mimetic of the findings of Hu et al. [30], who found that, without a GGGGS(x3) hinge, antigens did not bind antibodies with sufficient affinity for infection diagnosis. GGGGS(x3) hinges introduces six serine residues into a protein, explaining the predicted instability of flexible chimeras (Fig. 8a). Direct ligand-CBM interactions, as observed in Fig. 3, are suggestive of stable binding [50], whilst glycine AAs increase stability [56]. With 32 glycine residues introduced via flexible hinges, these chimeras are likely stable, however this requires in vitro confirmation.

### Larger proteins generally possess greater binding and catalysis

The importance of protein length is signified by linear relationships with binding affinity (Fig. 7g), catalytic activity (Fig. 6g), and stability (Fig. 8g). The two laccases that demonstrated visual CBM-ligand interaction upon addition of the flexible hinges, *F. venenatum* and *S. acidaminiphila* (Fig. 3), were also the largest proteins (672AA and 617AA, respectively). It is tempting to postulate that these hinges affected smaller proteins to a lesser degree due to tighter conformations, thus potentially requiring greater linker flexibility/length to reach nearer binding sites. This supports the lower binding affinities reported for both *S. agaradhaerens* (276AA) and *Stenotrophomonas* sp. (256AA) laccases. Indeed, increasing protein length seemingly improves binding affinity (Fig. 7g) and catalytic activity (Fig. 6g), as has been observed previously [56]. Whilst addition of flexible hinges increased linker length ~ 2-fold (Fig. 2), research suggests extending proteins > 4-fold may further enhance CBM binding potential [56]. It could be postulated that, owing to correlations observed between binding site surface area, catalytic activity, and binding affinity (Fig. 5e), that larger proteins may possess larger binding pockets and thus permit strong binding affinity and consequent catalysis.

### Aromatic AAs influence binding, catalysis and stability

Aromatic AAs were investigated due to the hypothesized hydrophobic interactions facilitating CBM-PE binding [28]. Our results supported this hypothesis, with greater aromatic AA numbers/composition yielding improved catalytic activity (Fig. 6e, f) and binding affinity (Fig. 7e, f), the former likely a result of the latter. This is substantiated by the high prevalence of hydrophobic residues predicted to directly bind dodecane, whilst increased intra-protein hydrophobic interactions likely explain the increased stability predicted (Fig. 8e, f). Notable limitations of this relationship were observed when proteins contained > 61 aromatic AAs, reporting reduced catalytic activity (Fig. 6e) and stability (Fig. 8e). Whilst incompletely understood, this phenomenon may derive from an overabundance of hydrophobic interactions impacting protein folding.

### PE binding and oxidation is influenced by bond type and abundance

Both hydrogen and disulphide bonds were investigated for their role in protein stability. Indeed, both cysteine composition (Fig. 8j) and hydrogen bond formation (Fig. 8h) improved stability predictions. The impact of disulphide bonds on other enzyme parameters, however, is complicated by non-linear, bimodal patterns. Whilst they are reported to influence both catalysis (Fig. 6i) and binding (Fig. 7i) to some degree, larger sample sizes are necessary to discern the relationship, with one group consisting of only four samples (Fig. 7i). On the other hand, reiteration of this pattern between variables supports an underlying influence of disulphide bonding. Understanding this phenomenon would permit stability, and thus binding affinity and catalysis, optimisation through cysteine residue modification. Further, this could be carried out in silico with adequate sample sizes, for rapid explorative analysis. As described for hydrogen bonding, greater covalent and VDW interactions improved protein stability, as has been previously suggested [31]. However, detrimental impacts above a threshold demonstrated a similar, non-linear, influence akin to that of disulphide bonding and hydrophobic interactions. This may be attributable to an overabundance of intra-protein bonding, occluding binding sites and manifesting as reduced substrate affinity and catalysis. The prevalence of this phenomenon within biomedical sciences, often termed “The Goldilocks Principle/Phenomenon”, suggests these findings may reflect the importance of balance. Despite a relatively robust sample size, this study suffers some obvious limitations. For example, inadequate sample size of certain groups limited data interpretation, particularly as > 30 samples are necessary to discern statistical significance with desirable effect size. This likely explains some of the variation observed within figures, along with the variation within bins; for example, the grouping of different laccases bearing the same CBM domain into a single bin. This is best evidenced by relatively smaller SEM in graphs separating the laccases into individual bins (panel “d” in Figs. 6, 7, 8 and 9). The overarching limitation to this study is the absence of in vitro translation and thus confirmation of polyethylene oxidation. Therefore, these predictions, regardless of robustness, require confirmation through further in silico and in vitro experiments.

### Future recommendations

It is necessary to confirm these predictions in vitro. Specifically, a thorough investigation into the oxidation potential of *S. acidaminiphila* MCO1 CBM2 chimera is required to understand the potential of industrial PE oxidation for large-scale microbial biodegradation. Translation of these findings into the laboratory is currently being assessed by our group. As a genetically engineered enzyme, biosafety considerations need assessed; however, the modifications proposed are not expected to enable persistence outside of controlled conditions. The influence of disulphide bonding on enzyme kinetics also requires clarification. An in-silico approach with sufficient sample size would permit a relatively simple explorative study to be performed, examining constructs with varying cysteine modifications. This would greatly advance the optimisation possibilities of exoenzymes and therefore increase their biotechnological value and consequent use within industrial biodegradation applications. This is supported by recent research suggesting cysteine substitution mutations may promote enzyme stability [32]. The high-throughput, rapid, inexpensive workflow used in this study can be adapted to engineer other exoenzymes. Indeed, esterase, lipase, and hydrolase upregulation in *Stenotrophomonas* sp. suggests these oxidative enzymes may play a role in the biodeterioration of PE hydrocarbons. These findings have been corroborated within recent literature [26], in addition to the upregulation of dehydrogenases, cutinases, and peroxidases. Hwang et al. [25] similarly found lipases to mediate PET degradation. It is therefore of interest to explore other exoenzymes for degradation of diverse plastic pollutants. Additionally, it is currently unknown how, or if, such exoenzymes are regulated; and if so, how plastics within the microenvironment may stimulate expression. It is possible that PE presence stimulates exoenzyme expression via regulatory systems, such as sigma/antisigma found upstream of the *Stenotrophomonas* sp. laccase. Elucidating such mechanisms would aid in understanding and consequently engineering microbes for more efficient petrochemical utilisation. The clear advantage of CBM2 chimeras necessitates the need to perform large-scale bioinformatic screening for optimal CBM2 candidates, akin to that performed in this study. This is expected to be a more demanding task, with > 22,000 CBM2 domains within the UniProt database under the PS51173 PROSITE identifier. Other type A CBMs should also be explored, with both CBM1 and CBM2 families displaying beneficial hydrophobic PE binding. Indeed, CBM3 domains of *Clostridium* and *Bacillus* sp. have demonstrated both cellulose [48] and PET [25] binding, with suggested improvement over CBM1 chimeras [48]. The importance of linkers suggests further optimisation potential. Site-directed substitution mutations would permit the addition of cysteine [32] and proline [56] residues to increase protein stability, whilst glycosylation of flexible hinge serine residues is proposed to reverse their destabilising properties [56]. Furthermore, native CBM linkers are proposed to improve flexibility [28] owing to reduced steric hinderance. With both C- and N-terminal native CBM positioning, this introduces the potential for dual chimeric exoenzymes. Such organisations are common within native endo-1,4-β-xylanases and have been proven efficacious within chimeric cellulases [24]. Dual chimeras can be readily examined using the workflow of this study, with the recommendation to screen C- and N-terminal type A CBM domains for candidate selection. Finally, although recombinant enzymes were the focus of this study, alternative whole-cell oxidation provides the benefit of biofilm formation. Indeed, isolates demonstrating greater biofilm capabilities are more effective at adhering to inert, hydrophobic PE surfaces [22], with concurrently higher biodegradation rates [57]. As PE oxidation is influenced to a greater extent by exoenzymes [33], whole-cell methods are not a necessity, but may provide the advantage of constitutive chimeric laccase expression, with consequent longevity and potentially cost-effectiveness, improving the realistic implementation of this technology for industrial-scale PE bioremediation.

## Conclusion

Reducing plastic pollution will have enormous impact on the health and wellbeing of both terrestrial and marine flora and fauna and is thus of primary interest to the scientific community and general public alike. In this study, optimisation of laccases for the degradation of polyethylene has been thoroughly investigated regarding the use of CBM domains to enhance binding and oxidation. Particularly, CBM2 domains were found to be significantly more advantageous than their CBM1 type A counterparts utilised within prior research. It is believed that this is the first study to elucidate binding of PE by CBM domains. These predictions suggest high potential of these laccase chimeras, with the incorporation of flexible hinges offering further optimisation potential to increase PE binding and thus oxidation. The novel laccase/MCO, MCO1, identified within *S. acidaminiphila*, is a clear candidate for proposed industrial PE oxidation, with further optimisation available to ensure optimal and rapid PE oxidation. It is advancements such as these that are necessary steps towards the implementation of such technologies within an industrial, large-scale setting. The introduction of a viable plastic recycling method is a pressing issue demanding an immediate resolution. Current methods, such as pyrolysis and land fill, are not sustainable for the reduction of anthropogenic impacts on the global climate. The implementation of this technology will bypass current concerns surrounding microbial plastic biodegradation, with slow preliminary oxidation optimised through improved binding and catalysis. Furthermore, strong putative binding at room temperature suggests improvements over pyrolysis through reducing energy demands and thus satisfying sustainability concerns surrounding high-temperature industrial processing. This will enable large-scale plastic oxidation and consequent microbial assimilation and mineralisation. It is reasonable to postulate that this technology may also be linked to other biotechnological processes, with oxidised plastics feeding into the production of industrially and pharmaceutically important products, such as antibiotics and biosurfactants. This is a significant step towards the realisation of a circular economy and more sustainable utilisation of petrochemicals. Future work must build upon these findings by screening the CBM2 family for candidate chimeras, with the expectation that the candidate enzyme in this study can be further improved through selection of an optimal CBM2 domain. Further optimisation is recommended, particularly the investigation of dual chimeras containing native C- and N-terminal type A CBM domains. It would be of interest to the field to consider other CBM domains as there are clearly significant differential PE binding between the CBM families. Specifically, it is advised that other type A CBM families undergo high throughput bioinformatic screening, akin to that within this study, such as the CBM 3, 5, and 10 families. Finally, is it imperative that these findings are confirmed in vitro.

## Methods

### Sequence information

Laccase DNA sequences were obtained from the National Centre for Biotechnology Information (NCBI) for *F. venenatum* (GenBank: CEI66207), *Stenotrophomonas acidaminiphila*, *Salipaludibacillus agaradhaerens* (GenBank: WP_078579868), and *Stenotrophomonas* sp. The linker sequence was derived from *Trichoderma reesei*, as described by Santacruz-Juarez et al. [38] (UniProt Accession: P62694). CBM1 sequence data is available at the accession identifiers stated within Fig. 1 and were obtained from UniProt. Laccase candidate sequence information is available within Additional file 2.

### Candidate laccase selection

The *S. acidaminiphila* laccase was selected using standalone BLASTp [34]. The query sequence was obtained from Zampolli et al. [27] supplementary data. *S. acidaminiphila* genome was annotated through Rapid Annotation using Subsystem Technology (RAST) pipeline (version 2.0) [35]. This provided predicted coding sequences (CDSs) which supplemented an AA database for the BLASTp search. An E-value cut-off of 10^− 5^ (1e-5) was applied and a query-coverage score (%) determined to assess hit quality: (identity (%) x query coverage) / 100. This was also applied to identify the candidate laccases from *Stenotrophomonas* sp. and *S. agaradhaerens*, organisms being investigated within our group. The *F. venenatum* candidate was selected from 13 potential laccases encoded in the genome, via molecular docking.

### Candidate CBM1 selection

105 manually curated CBM1 domain sequences were retrieved from UniProt SwissProt database [36] using the PROSITE [37] identifier “PS51164”, retrieved from the Carbohydrate-Active Enzymes (CAzy) database [58]. A CBM1 heatmap was generated using molecular docking data and ggplot2 (version 3.3.5) [59] and ggdendro (version 0.1.23) packages in R. Molecular docking data can be found in Additional file 3.

### Molecular docking

WT and chimeric sequences were constructed in a text-editor and submitted in FASTA format to Robetta [60] for ab initio protein modelling using RoseTTAFold algorithm and default parameters. RoseTTAFold was selected as the optimal algorithm to provide high quality, high-throughput, and more reliable 3D structure prediction compared to more imprecise alternatives, guided by its performance within recent Critical Assessment of Techniques for Protein Structure Prediction (CASP) trials [61]. Necessary within ab initio modelling, 3D structures were validated based on provided GDT confidence scores rather than similarity to a curated homologous template. Confidence scores above 0.5 (between 0 and 1) were considered acceptable for downstream analyses. This score was used to determine the most appropriate model from the resulting five provided by Robetta. Resulting PDB files were re-formatted, as PDB files, within OpenBabelGUI (version 3.1.1) [39]. This was necessary for ab initio model compatibility within docking software. Dodecane (C_12_H_26_) and eicosane (C_20_H_42_) 3D models were obtained through converting SMILES format into mol2 filetype. Molecular docking was performed in AutoDock Tools (version 1.5.7) [62] using AutoDock Vina algorithm (version 1.2.0) [63]. Ligand mol2 files were prepared within AutoDock Tools using the Ligand > Input selection to open each ligand and then output as a PDBQT file for consequent docking. Gasteiger charges [64] were added to ligands automatically to prepare Gasteiger/Kollman charges for protein-ligand docking simulations. Receptor/laccase WT and chimeric PDB files were opened in AutoDock Tools and processed via the following: merge non-polar hydrogens; add polar hydrogens; add Kollman charges; and checking the total charges on residues to properly distribute charges. Blind docking was utilised to prevent restrained docking to a predicted active site thus permitting more diverse binding interactions. Grid box parameters were maximised (X = 120; Y = 120; Z = 120; 1Å spacing) and positioned to cover the entire enzyme model. These data were utilised to construct a config file for each enzyme docking simulation. Molecular docking was performed using AutoDock Vina algorithm through the Windows Command Prompt (CMD), utilising a Perl script to characterise the binding configuration, receptor, and ligand(s). Gibbs’ free energy (ΔG, kcal mol^− 1^) and Root-Mean-Square Deviation (RMSD) output were noted from the CMD whereas individual ligand pose PDBQT files were stored for consequent 3D modelling of protein-ligand interactions. The dissociation constant (Kd) was calculated to estimate ligand-binding affinity using the molecular docking ΔG output, using the following formula: Kd = e^(-∆G/(R×T)); where e^ = the exponent; ΔG = Gibbs’ free energy in joules mol^− 1^; R = the Gas Constant (8.314); and T = temperature in Kelvin. Approximate room temperature, 298 K, was chosen for Kd calculations and binding affinity was expressed in micromolar (µM) units. Output ligand pose and enzyme PDBQT files from each protein-ligand interaction were compiled within Pymol (version 2.5.3) [65] and exported as a PDB complex. This served as input for both Discovery Studio (DS) (version 21.1.0.20298) [66] and ChimeraX (Version 1.4) [67]. The ligand was defined in DS and 2D interactions mapped to inform enzyme binding residues and interaction types. ChimeraX was used to generate 3D models of protein-ligand interactions, permitting annotating of chimeric regions and binding residues for visualisation. Catalytic activity (k_cat_) was predicted using DLk_cat_ algorithm [40] with default parameters and substrates in SMILES format. Guaiacol was used for pilot experimentation to estimate algorithm reliability whilst pooled dodecane and eicosane K_cat_ values were used within correlation and univariate ANOVA analyses. The distance between ligand and CBM domains was calculated within Pymol using the following command: “distance (name of new object), ////(name of ligand), ////(residue number)/CA. For example: distance d1, ////UNL, ////727/CA, where CA is the alpha carbon of the 727th AA residue. This returned all distances in angstrom (Å) between the alpha carbon of the AA residue and each carbon atom of the ligand. Dashes were removed within Pymol and an image taken of the total distances. This was converted into text format using Microsoft OneNote built-in optical character recognition (OCR) tool and averaged using Microsoft Excel. Similarly, disulphide bond number was manually assessed within Pymol by selecting Show > Disulphides > Lines and counting the number of disulphide bonds occurring in the protein-ligand complex. Pymol was also utilised for calculating estimated binding site surface area (Å^2^) by selecting Action > Compute > Surface Area > Molecular. Binding sites were defined from examination of 2D interaction residues within DS as aforementioned. Aromatic AA number and composition variables were obtained from inspection of AA sequences using ExPASy ProtParam, attaining both the number and composition % of tyrosine, phenylalanine, and tryptophan residues. This tool also predicted protein stability, hydrophobicity, and cysteine composition. Protein stability was predicted via an instability index as described by Guruprasad et al. [41] which considers protein length and the presence of AA residues prevalent in unstable proteins (methionine, glutamine, proline, glutamic acid, and serine). Aliphatic index, reflecting thermostability and hydrophobicity, was assessed using the formula proposed by Ikai [42], considering the presence of alanine, valine, isoleucine, and leucine aliphatic side chains. GRAVY reported within ProtParam represented protein hydrophobicity, with higher values reflecting greater hydrophobicity [43] based on hydrophobic AA side chain prevalence. Hydrogen bonds were enumerated within DS, via Structure > Monitor > HBonds, and output as “Total Favorable Non-Bonds” within the Data Table. Data from molecular docking, catalysis predictions, and protein characteristics can be found in Additional file 4.

### Statistical analyses

Sensitivity, *a priori*, and *post hoc* power analyses was performed to determine effect size, sample size requirements, and attained power of computational data, respectively. Microsoft Excel was used for data entry and management, and the generation of figures. SPSS was used for univariate ANOVA, bivariate correlation, PCA, means testing, descriptive statistics analyses, and for the generation of figures. SEM was displayed as error bars. Bivariate correlation was performed to investigate pairwise correlation between dependent variables to examine potential associations. This was analysed and plotted using corrplot package (version 0.92) [68] in R. Univariate ANOVA was performed within SPSS to investigate the inter-group variability of independent variables on a single dependent variable. This was repeated for several dependent variables owing to computational limitations performing multivariate correlation analysis. *Post hoc* Tukey’s testing was performed following univariate ANOVA to discern specific, pairwise, inter-group differences. Probability (p) values of < 0.05, < 0.01, and < 0.001 discerned statistically significant (*), highly statistically significant (**), and very highly statistically significant (***) differences between groups, respectively.

## Supplementary Information

**Additional file 1. **Experimental kcat Comparison.

**Additional file 2. **Sequence data.

**Additional file 3. **Molecular Docking list of CBM1 domains.

**Additional file 4. **Construct Docking data.

## Data Availability

The datasets generated and/or analysed during the current study are available in the National Center for Biotechnology Information (NCBI) repository and/or the UniProt repository, with accession numbers provided within Additional File 2. Generated data are available within the Supplementary Files.

## Abbreviations

AA: Amino Acid
ANOVA: Analysis of Variance
BLASTp: Protein Basic Local Alignment Search Tool
CAzy: Carbohydrate-Active Enzymes
CBM: Carbohydrate-Binding Module
CDS: Coding Sequence
CMD: Command Prompt
DLk_cat_: Deep-Learning k_cat_
DS: Discovery Studio
GRAVY: Grand Average of Hydropathy
HDPE: High-Density Polyethylene
k_cat_: Catalytic constant
Kd: Dissociation constant
LDPE: Low-Density Polyethylene
MaPs: Macroplastics
MCO: Multicopper Oxidase
MHET: Mono (2-hydroxyethyl) Terephthalate
MPs: Microplastics
NCBI: National Center for Biotechnology Information
NPs: Nanoplastics
OCR: Optical Character Recognition
PCA: Principal Component Analysis
PE: Polyethylene
PET: Polyethylene Terephthalate
qPCR: Quantitative PCR
RAST: Rapid Annotation using Subsystem Technology
RMSD: Root-Mean-Square Deviation
SEM: Standard Error of the Mean
VDW: Van der Waals
ΔG: Change in Gibbs Free Energy
