# Supplementary material for: A computational approach to optimising laccase-mediated polyethylene oxidation through carbohydrate-binding module fusion
Source: BMC Biotechnol. 2023 Jul 6;23:18. doi: 10.1186/s12896-023-00787-5 (PMC10324223; doi:10.1186/s12896-023-00787-5)
Supplement: Supplementary file 2 — Additional file 2. Sequence data. [file 12896_2023_787_MOESM2_ESM.docx]

**Additional file 2 – Sequence data**

| **Component** | **NCBI Accession** | **UniProt Accession** |
| --- | --- | --- |
| **Laccases** |  |  |
| *Fusarium venenatum* laccase | CEI66207 | A0A2L2T4Y8 |
| *Stenotrophomonas acidaminiphila* “MCO1” laccase | WP_182335395 | ns |
| *Stenotrophomonas acidaminiphila* “MCO2” laccase | KRG86672 | A0A0R0DY51 |
| *Salipaludibacillius agaradhaerens* laccase | WP_078579868 | ns |
| *Stenotrophomonas* sp. laccase | WP_049437910 | ns |
|  |  |  |
| **Carbohydrate-binding modules (CBMs)** |  |  |
| A2R5N0 CBM1 domain | CAK42466 | A2R5N0 |
| A1C4H2 CBM1 domain | EAW15312 | A1C4H2 |
| B8NIV9 CBM1 domain | EED50165 | B8NIV9 |
| Q5BCX8 CBM1 domain | CBF85202 | Q5BCX8 |
| A0A1D8EJG8 CBM1 domain | AOT21131 | A0A1D8EJG8 |
| P62694 CBM1 domain | CAA49596 | P62694 |
| CBM2 domain (*S. cellulosum*) | CAN99125 | A9G955 |
|  |  |  |
| **Linkers** |  |  |
| *T. reesei* linker sequence (for chimeras, between laccase and CBM domain) | CAA49596 | P62694 |
| *S. cellulosum* linker sequence (for CBM2 laccase chimeras) | CAN99125 | A9G955 |

**ns = not submitted within the relevant database**
